# To What Extent is Drinking Water Tested in Sub-Saharan Africa? A Comparative Analysis of Regulated Water Quality Monitoring

**DOI:** 10.3390/ijerph13030275

**Published:** 2016-03-02

**Authors:** Rachel Peletz, Emily Kumpel, Mateyo Bonham, Zarah Rahman, Ranjiv Khush

**Affiliations:** 1 The Aquaya Institute, Nairobi 00505, Kenya; emily@aquaya.org (E.K); mateyobonham@gmail.com (M.B.); 2 The Aquaya Institute, Larkspur, CA 94939, USA; zarah.rahman@gmail.com (Z.R.); ranjiv@aquaya.org (R.K.)

**Keywords:** water monitoring, water quality, drinking water, regulated testing, fecal contamination, sub-Saharan Africa, water utilities, health agencies, institutional performance

## Abstract

Water quality information is important for guiding water safety management and preventing water-related diseases. To assess the current status of regulated water quality monitoring in sub-Saharan Africa, we evaluated testing programs for fecal contamination in 72 institutions (water suppliers and public health agencies) across 10 countries. Data were collected through written surveys, in-person interviews, and analysis of microbial water quality testing levels. Though most institutions did not achieve the testing levels specified by applicable standards or World Health Organization (WHO) Guidelines, 85% of institutions had conducted some microbial water testing in the previous year. Institutions were more likely to meet testing targets if they were suppliers (as compared to surveillance agencies), served larger populations, operated in urban settings, and had higher water quality budgets (all *p* < 0.05). Our results indicate that smaller water providers and rural public health offices will require greater attention and additional resources to achieve regulatory compliance for water quality monitoring in sub-Saharan Africa. The cost-effectiveness of water quality monitoring should be improved by the application of risk-based water management approaches. Efforts to strengthen monitoring capacity should pay greater attention to program sustainability and institutional commitment to water safety.

## 1. Introduction

Exposure to microbial pathogens that contaminate water supplies is responsible for a significant fraction of the public health burden faced by low-income countries [1,2]. Unsafe drinking water is a major cause of diarrhea, which is responsible for an estimated 10% of the global mortality among children under the age of five [2,3]. Viral and parasitic infections, environmental enteropathy, and undernutrition are other health concerns associated with unsafe water [2]. Consequently, information about drinking water quality is essential for guiding efforts to reduce waterborne illnesses: reliable, up-to-date water quality data can identify high-risk water sources, determine effective water treatment methods, and contribute to the evaluation of water and sanitation improvement programs. For many low-income countries, institutional responsibilities for regulated water quality monitoring are well-established by national and regional laws or guidelines [4,5]. These responsibilities generally fall into two categories: (1) operational monitoring (or water quality control) by water suppliers and (2) surveillance (or compliance) monitoring by an independent agency.

Regular operational monitoring by water suppliers is a key tool for maintaining treatment process control and verifying water quality [6]. For the purposes of this paper, we define water suppliers (also commonly called water providers or utilities) as regulated institutions responsible for treating and monitoring water supplies distributed through piped systems. According to World Health Organization (WHO) Drinking Water Quality Guidelines, suppliers should frequently conduct operational monitoring, often limited to a set of critical parameters such as pH, residual chlorine, turbidity, and indicator bacteria [6,7]. Ideally, operational monitoring triggers immediate corrective actions when test results indicate that a water supply system is compromised.

Surveillance monitoring requires an agency, usually responsible for public health and independent from direct water provision, to assess whether drinking water sources meet national standards [6,7]. In addition to water quality testing, the WHO Guidelines recommend that surveillance monitoring includes a comprehensive evaluation of the adequacy of water sources, including water quantity, accessibility, affordability, and continuity [6,7,8]. Drinking water surveillance is, in principle, linked to the oversight of water supplies and to resource allocations for improving water quality.

Despite these established responsibilities, water quality monitoring in low-income countries is often constrained by poor regulatory enforcement and insufficient financial, human, and logistical resources [4,5,7,9]. This study describes the current status of regulated microbial water quality monitoring in sub-Saharan Africa. Our objectives were to: (i) document existing data and performance levels for operational and surveillance water quality monitoring across a range of institutions and countries and (ii) identify institutional and contextual factors that may affect monitoring performance.

## 2. Materials and Methods

### 2.1. Country Selection and Study Institutions

**Figure 1:**
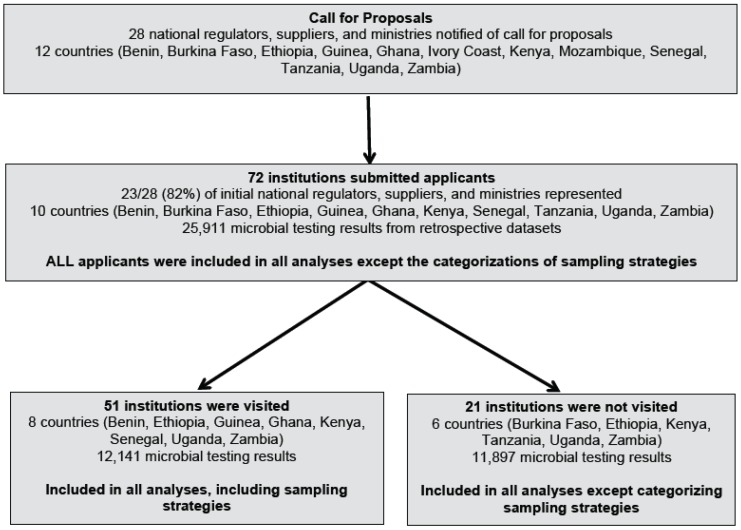
Recruitment process for institutions (water suppliers and surveillance agencies) to participate in the Monitoring for Safe Water research initiative.

This study was conducted under The Aquaya Institute’s Monitoring for Safe Water (MfSW) research initiative to build water safety monitoring and management capacity in sub-Saharan Africa [10]. In collaboration with the WHO, International Water Association (IWA), and African Water Association (AfWA), we identified twelve African countries that encompass the economies, geographies, and water management institutional frameworks [5] in sub-Saharan Africa: Benin, Burkina Faso, Ethiopia, Ghana, Guinea, Ivory Coast, Kenya, Mozambique, Senegal, Tanzania, Uganda, and Zambia. We then issued a call for applications, open from December 2012 to May 2013, from regulated institutions responsible for operational or surveillance monitoring in these countries for participation in MfSW; institutions without clearly defined regulatory requirements for water quality monitoring, such as private water vendors and non-governmental organizations, were not eligible to apply. The proposals were the first step of assessing eligibility for receiving MfSW funding resources; this incentivized institutions to submit applications. The data used in this study was collected from these proposals prior to institutional participation in MfSW. The call for proposals was disseminated to national regulators, suppliers, and ministries of water and health (Figure 1).

### 2.2. Data Collection

The applications served as a baseline survey of regulated water quality monitoring. Institutions described their current water quality testing activities (e.g., testing frequencies, testing methods, geographical coverage, sampling plans, staffing structure) and provided one year of retrospective raw (not summarized) microbial water quality data (which we refer to as retrospective datasets), if available. We conducted in-person, semi-structured interviews with water quality managers from a subset of institutions (71%, 51/72) that included a range of institutional types (supplier/surveillance), sizes, and geographical locations. We used the in-person interviews to evaluate sampling strategies (using open-ended questions) and to obtain missing data. Applicants that we did not visit (29%, 21/72) were unresponsive or in challenging geographical locations; however, the only information that we did not collect from unvisited institutions was data on sampling strategies.

Information from the written application and the water quality data were entered into Excel and analyzed using Stata 12 (StataCorp LP, College Station, TX, USA) or R (r-project.org) software packages (R Foundation for Statistical Computing, Vienna, Austria). Interviews were entered and coded using NVivo Beta for Mac and NVivo 10 for Windows (QSR International (Americas) Inc., Burlington, MA, USA).

### 2.3. Retrospective Datasets

We used the number of microbial tests conducted by each institution as a key outcome variable to measure institutional testing performance, defined as the number of tests conducted per unit time compared to WHO Guidelines or national standards. This metric of performance examines the extent to which testing was conducted; our analysis of water quality data and compliance (*i.e.*, percentage of samples absent of fecal contamination) will be presented in a separate publication [11]. Data on number of tests were collected through self-reports in the written application, and verified using retrospective microbial water quality datasets. We included tests for all fecal indicator organisms: *Escherichia coli (E. coli),* thermotolerant (fecal) coliforms), *Streptococci*, *Enterococci*, and hydrogen-sulfide (H_2_S) producing bacteria in our calculations of test numbers.

Though 48 institutions provided retrospective datasets, we excluded 15 from our analysis of institutional testing performance, either because they only provided summary statistics (*i.e.*, percentage of tests passed or failed) or because the data was older than 12 months (Table 1).

We screened for potentially fictitious results in the retrospective datasets by determining if the distribution of the leading digit of included parameters fit Benford’s law [12,13] using visual inspection and χ^2^ tests [11]. Our analysis included 55,567 test results from 35% (41/116) of the laboratories, which represented datasets from 67% (23/34) of institutions that provided retrospective data. Differences of *p* < 0.01 were classified as potentially suspect, representing 2% of the datasets that could be examined with Benford’s law. More details are provided in the Supplementary Materials (Figure S1).

### 2.4. Monitoring Performance and Data Analysis

We used the WHO Guidelines for Drinking Water Quality, 4th edition [6] and national standards (Table S1, [14,15,16,17,18,19,20,21,22,23,24]), where available, as benchmarks for evaluating each applicant’s monitoring performance with respect to the number of microbial water quality tests conducted. Because we were uncertain about the completeness of the retrospective water quality datasets, we used reported testing numbers to evaluate institutional monitoring performance (dataset numbers were lower than reported numbers for 76% of institutions, though 88% of institutions were within one order of magnitude (Figure S2)). We analyzed sampling strategies by asking for institutional methods in the written applications and interviews and by obtaining water source type information (*i.e.*, piped network, household storage, *etc.*) from the retrospective microbial water quality datasets, when available.

### 2.5. Institutional Characteristics and Data Analysis

The application information included the following characteristics: institution type (supplier or surveillance), population served, area of jurisdiction, number of connections (for suppliers) or water sources (for surveillance), years in operation (for suppliers), water quality budget, and staffing levels. Additionally, to determine whether the applicants operated in urban or rural settings, we used country-level definitions derived from national-level census data [25]. We defined “independent regulation” as the presence of a regulatory oversight agency that is independent of the agencies responsible for services provision and whose primary role is water regulation.

Covariates were cross-tabulated with performance (*i.e.*, meeting WHO Guidelines) and compared using Χ^2^ tests. For analyses that included only a subset of applicants (e.g., suppliers or surveillance only) and small sample sizes, we used Fishers Exact Test [26].

### 2.6. Ethical Approval

The study protocol was submitted to the Western Institutional Review Board (WIRB) (Olympia, WA, USA) for ethical review and received a determination of exemption from full review under 45 CFR 46.101(b)(2) of the Common Rule. Institutions agreed to share their water quality monitoring information (including raw data) as part of the MfSW application process. 

## 3. Results

### 3.1. Study Institutions

The MfSW initiative received applications from 72 regulated institutions spanning 10 countries: Benin, Burkina Faso, Ethiopia, Ghana, Guinea, Kenya, Senegal, Tanzania, Uganda, and Zambia (Figure 1). These applicants represented 82% (23/28) of the national regulators, suppliers, or ministries that were asked to disseminate the MfSW call for applicants (4/5 of remaining institutions were from Mozambique or the Ivory Coast). The applicants included 37 suppliers, representing various organizational structures ranging from small-scale private water supply operators to national-level government corporations (Table 2). All suppliers were located in urban areas and most served medium or small populations (84% served <500,000 people). Approximately two-thirds (62%) were independently regulated. The remaining 35 applicants were surveillance agencies, including national-level health and water Ministries, laboratories affiliated with these ministries, and District Health or Water Offices (Table 2). 

Most of the surveillance agencies were District Health or Water Offices (69%), most were located in rural areas (77%), and most were responsible for serving large populations (94% covered >100,000 people). None of the surveillance agencies were independently regulated. Most surveillance agencies monitored both piped and point water sources in their areas of jurisdiction (88%, data missing for two institutions). Additional institutional characteristics are described in Table 2.

### 3.2. Sampling Strategies

The retrospective datasets included analysis of 22,957 water samples collected by suppliers and 1081 samples collected by surveillance agencies (Table 3). The majority of samples (67%) from suppliers were taken from consumer taps in the piped distribution system, with the remaining samples from other sections of the distribution system (source water and water treatment plants) (Table 3). Surveillance agencies tested a range of water sources, though the largest fractions were consumer taps in piped networks (31%) and storage containers within households or institutions (15%) (Table 3).

We obtained information on sampling strategies for 32/72 institutions (Table 4). Suppliers most commonly sampled along the distribution system, including raw water, treatment plant water, and piped network water (10/19 suppliers). Surveillance agencies most commonly had a reactive strategy, such as sampling after a disease outbreak (7/13 surveillance agencies).

### 3.3. Testing Methods

Almost all institutions (99%) reported some type of water quality monitoring activity (physical, chemical, and/or microbial), either within their institution, through an external laboratory, or both (Table 5). Eighty-five percent of institutions reported some microbial testing in the past year, and most relied upon *E. coli* (24%), thermotolerant (fecal) coliforms (36%), and/or total coliforms (29%) as indicators of fecal contamination. A few suppliers also monitored fecal *Streptococci* (*n* = 4), *Clostridium perfingens* (*n* = 3), *Enterococci* (*n* = 1), and heterotrophic bacteria (*n* = 8). One supplier and two surveillance agencies tested for H_2_S-producing bacteria. Among microbial testing methods, membrane filtration was the most common (46%), though presence-absence (25%), most probable number (MPN) (15%), and 3M**™** Petrifilm**™** (3M, St. Paul, MN, USA) methods (4%) were also used (Table 5).

### 3.4. Monitoring Guidelines and Standards

Most of the countries represented in this study possess national drinking water standards that specify microbial testing requirements, including the number of samples that must be analyzed over a given period, indicator species, and reporting formats. These national standards are commonly derived from the WHO Guidelines. Operational and surveillance monitoring requirements for formally managed water supplies are generally based on the size of the population served (such as one sample per month for supplies serving <5000 people, as recommended by the WHO). Surveillance monitoring requirements for informal supplies and point sources are often based on the number of water sources within the agency’s jurisdiction (such as monitoring of all point sources every 3–5 years, as recommended by the WHO) [6]. Burkina Faso, Guinea, and Senegal, do not have national standards but rely upon WHO Guidelines to determine operational and surveillance monitoring activity [15]. In addition, Zambia has not defined national standards for monitoring non-piped sources [23].

### 3.5. Monitoring Performance

**Figure 2:**
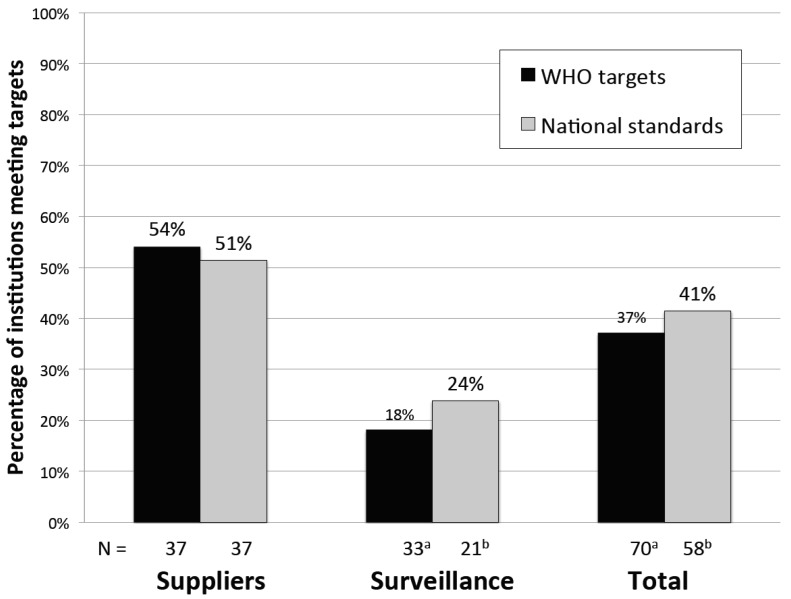
Monitoring performance over one year with respect to WHO Guidelines and national standards for suppliers and surveillance agencies. Performance in meeting WHO Guidelines is in black while performance in meeting national standards is in gray. **^a^** Data is missing for two surveillance agencies; **^b^** Twelve surveillance agencies do not have national standards (Zambia [23]), data missing for two additional institutions.

According to two benchmarks for testing performance, most institutions did not conduct enough microbial water quality tests in one year: only 37% (26/70) of institutions met WHO Guidelines and only 41% (24/58) of institutions met national standards (Figure 2). Performance was similar when measured against either WHO Guidelines or national standards because many countries derive their national standards from WHO Guidelines, as discussed above. A higher percentage of suppliers than surveillance agencies conducted sufficient tests (Figure 2), and in aggregate, suppliers conducted more microbial tests per year than surveillance agencies (mean of 1731 *vs.* 196, *p* = 0.01) (Table 5).

### 3.6. Factors Associated with Monitoring Performance

Suppliers were significantly more likely than surveillance agencies to meet WHO Guidelines for the number of microbial tests conducted in one year (18% surveillance *vs.* 54% suppliers, *p* < 0.01) (Table 6). Larger suppliers were more likely than smaller suppliers to meet WHO Guidelines based on population served, area of jurisdiction, and number of connections. Additionally, West African suppliers (9/10) were more likely to meet WHO Guidelines than suppliers in other regions (11/27) (*p* = 0.01). Higher water quality budgets were also associated with better supplier performance: 80% of suppliers with a budget of at least U.S. $0.05 per person served met WHO Guidelines, compared to 24% with lower budgets (*p* < 0.01). Other institutional and regulatory environment characteristics (*i.e*., number of staff, years in operation, independent regulation, and documented national standards) were not significantly associated with supplier performance (*p* > 0.05) (Table 6).

Among surveillance agencies, population coverage of ≥500,000 was associated with a greater likelihood of meeting WHO Guidelines for the number of microbial tests conducted in one year (42% *vs.* 5% with populations <500,000, *p* = 0.02). However, the number of water sources was inversely associated with meeting WHO Guidelines (38% of those with <1000 sources compared to 5% with ≥1000 sources, *p* = 0.03). Furthermore, West African surveillance agencies (3/4) had better performance than those in other regions (3/29) (*p* = 0.01). Other institutional characteristics were not associated with monitoring performance by surveillance agencies (*p* > 0.05), though many categories had small sample sizes (Table 6).

## 4. Discussion 

This study provides a comprehensive analysis of regulated water quality monitoring in sub-Saharan Africa. Among 72 institutions spanning 10 countries, 85% reported microbial testing activity in the past year; however, most did not achieve the annual number of microbial water quality tests specified by their relevant national standards or the WHO Guidelines for Drinking Water Quality.

Suppliers were generally stronger performers than surveillance agencies, which may be related to their different economic structures. Suppliers typically receive revenue for supplying water, which can be used to support water quality testing activities, while surveillance agencies are generally reliant on government budget allocations and donor priorities. We found that supplier performance was associated with higher per-capita budgets for water quality monitoring and larger population sizes. Stronger surveillance agency performance was associated with two contradictory factors: large population coverage and monitoring of a small number of water sources (Table 6). This contradiction may be explained by the designation of piped distribution networks as a single source by some surveillance agencies. For both suppliers and surveillance agencies, operations in West Africa compared to other regions was associated with better performance; however, since the jurisdiction of all West African applicants was on a national level, better performance may be a function of larger size. Regulation by an independent authority was not associated with better supplier performance, and the presence of national standards did not improve either supplier or surveillance agency performance.

This study has several limitations. Primarily, the institutions that we evaluated were not selected randomly from a representative pool of regulated monitoring institutions in sub-Saharan Africa. Our dissemination of the MfSW call for proposals in collaboration with WHO, IWA, and AfWA may have biased the selection towards members of their networks. To examine potential selection bias in our study group, we compared a subset of the selected institutions with their national counterparts. In Kenya, for example, our applicants (suppliers and surveillance agencies) were from 8/47 counties; on average, consumer characteristics, including socio-economic status, education levels, and access to water and sanitation among these eight counties were comparable to national averages (Tables S2 and S3 [27,28]). Based on these types of analyses and the broad range of institutional characteristics and monitoring performance levels captured among our 72 applicants, we posit that our study captures the diversity of supplier and surveillance agencies found in non-fragile sub-Saharan African states (as defined by the World Bank [29]). 

Additional limitations include our non-random selection process for visiting monitoring institutions and our reliance on self-reported information. Where possible, we confirmed data during follow-up interviews and by examining retrospective testing datasets. Nevertheless, we were not able to confirm some information, such as the number of sources within a surveillance agency’s area of jurisdiction or whether the datasets were complete. Finally, we did not account for similarities (or clustering) between applicants when examining associations with monitoring performance: for example, in cases where we received applications from multiple urban suppliers operating under the same National Provider, we treated them as separate institutions in our analysis.

Despite these limitations, to our knowledge this is the first comprehensive study of regulated water quality monitoring in sub-Saharan Africa. Though most of the suppliers and surveillance agencies in our study did not achieve the microbial testing levels specified in the WHO Guidelines or national standards, their consistent efforts to engage in some testing activity indicates that the foundations for regulated water quality monitoring are largely in place in non-fragile states, which provides opportunities for strengthening the application of water quality data in water safety management. Our study also suggests that efforts to improve monitoring performance should address the constraints faced by weaker institutions. Among suppliers, stronger performance by larger institutions has been previously noted [9]; however, the majority of sector assistance still focuses on large systems [30], and capacity building programs such as Water Operators Partnerships (WOPS) in Africa are generally directed towards the big, visible suppliers that are easier to engage [31]. The majority of Africans, however, rely on rural and small urban water supplies that do not fall under the jurisdiction of large urban, regional or national suppliers [32,33]. Actions to decrease the performance gaps between small suppliers and public health offices in secondary towns and the large suppliers may prove more productive for realizing public health objectives.

Our finding that surveillance agencies in sub-Saharan Africa are more likely to struggle than suppliers in achieving monitoring requirements confirms observations in other regions [5]. Water quality testing is dominated by operational monitoring of urban piped systems (>95% of our water quality samples were from urban piped networks), while surveillance monitoring of non-piped sources is limited. This is a significant public health concern since surveillance testing is the only form of oversight applied to the informal water supplies and point sources that serve a vast majority of Africans: 32% of sub-Saharan Africans drink from unimproved sources, and an additional 52% drink from other sources that are not piped water to their premises [32]. Rural areas are less likely to have access to improved water and sanitation [32]; therefore, surveillance monitoring is particularly important for evaluating and addressing urban-rural inequalities. Surveillance agencies in low-income countries are handicapped by their relatively small budgets for water quality monitoring, their large jurisdictions, and their multiple public health responsibilities. 

## 5. Conclusions

This research found that regulated water quality monitoring activities in sub-Saharan Africa did not achieve testing levels specified by WHO Guidelines or national standards, particularly among smaller water suppliers and surveillance agencies. Our analysis shows that bureaucratic procedures such as the development of national standards and the creation of independent regulatory bodies are unlikely to alleviate these constraints without parallel commitments to implementation of these policies, such as greater resource allocations for monitoring in small towns and rural areas. In addition, surveillance programs must focus on improving the cost-effectiveness of water quality monitoring. Strategies to improve cost-effectiveness include the implementation of risk-management procedures [34,35], including applying sanitary surveys to reduce testing of clearly contaminated supplies and prioritizing water quality parameters that represent the greatest risks to public health. Improving the supply of testing equipment and consumables in rural settings will require better coordination between surveillance agencies and private sector equipment suppliers. Furthermore, institutions require the resources and skills to act upon testing results to improve water quality. Finally, capacity building of monitoring programs should focus on program sustainability and applying water quality data towards improved water safety.

## Figures and Tables

**Table 1:** Summary of retrospective microbial water quality test datasets. Institutions provided raw data rather than data summaries.

Dataset Characteristic	Category	Supplier (*N* = 37)	Surveillance (*N* = 35)	Total (*N* = 72)
Microbial retrospective datasets	Datasets provided	31 (84%)	17 (49%)	48 (67%)
	Dataset not provided	6 (16%)	18 (51%)	24 (33%)
Method datasets received	Emailed	25 (68%)	10 (29%)	35 (49%)
	Scanned data during visits	6 (16%)	7 (20%)	13 (18%)
	No datasets provided	6 (16%)	18 (51%)	24 (33%)
Format provided	Portable Document Format (PDF)	17 (46%)	8 (23%)	25 (35%)
	Microsoft Word	6 (16%)	4 (11%)	10 (14%)
	Microsoft Excel	4 (11%)	4 (11%)	8 (11%)
	Multiple formats	4 (11%)	1 (3%)	5 (7%)
	No datasets provided	6 (16%)	18 (51%)	24 (33%)
Included handwritten entries	Yes	9 (24%)	7 (20%)	16 (22%)
	No	22 (59%)	10 (29%)	32 (44%)
	No datasets provided	6 (16%)	18 (51%)	24 (33%)
Completeness	≥12 months of testing results	9 (24%)	1 (3%)	10 (14%)
	6–11 months of testing results	11 (30%)	3 (9%)	14 (19%)
	0–5 months of testing results	17 (46%)	31 (89%)	48 (67%)

**Table 2:** General characteristics of study institutions. Suppliers are regulated institutions responsible for providing water through managed piped distribution systems. Surveillance agencies operate independently of water suppliers and are responsible for ensuring the safety of drinking water services.

Institution Characteristic	Category	Suppliers (*N* = 37)	Surveillance (*N* = 35)	Total (*N* = 72)
Country	Benin	7 (19%)	1 (3%)	8 (11%)
	Burkina Faso	1 (3%)	1 (3%)	2 (3%)
	Ethiopia	4 (11%)	5 (14%)	9 (13%)
	Ghana	1 (3%)	0 (0%)	1 (1%)
	Guinea	1 (3%)	0 (0%)	1 (1%)
	Kenya	8 (22%)	5 (14%)	13 (18%)
	Senegal	0 (0%)	3 (9%)	3 (4%)
	Tanzania	2 (5%)	0 (0%)	2 (3%)
	Uganda	10 (27%)	8 (23%)	18 (25%)
	Zambia	3 (8%)	12 (34%)	15 (21%)
Institution Type	*Supplier*			
	National Supplier ^1^	15 (41%)	-	15 (21%)
	Provincial Supplier	3 (8%)	-	3 (4%)
	City/town Supplier	14 (38%)	-	14 (19%)
	Small-scale Private Water Supplier	5 (14%)	-	5 (7%)
	*Surveillance*			
	National Health Ministry ^1^	-	5 (14%)	5 (7%)
	National Water Ministry	-	1 (3%)	1 (1%)
	Regional Laboratory ^2^	-	5 (14%)	5 (7%)
	District Health or Water Office	-	24 (69%)	24 (33%)
Population Served ^3^	<100,000	13 (35%)	2 (6%)	15 (22%)
	100,000–<500,000	18 (49%)	18 (56%)	36 (52%)
	≥500,000	6 (16%)	12 (38%)	18 (26%)
Urban/rural	Urban	37 (100%)	8 (23%)	45 (63%)
	Rural	0 (0%)	27 (77%)	27 (38%)
Type of water sources in jurisdiction ^4^	Piped water only	37 (100%)	3 (9%)	40 (57%)
	Point sources only	0 (0%)	1 (3%)	1 (1%)
	Both point sources and piped water	0 (0%)	29 (88%)	29 (41%)
Number of connections (suppliers only) ^5^	<5000	9 (27%)	-	-
	5000–<10,000	8 (24%)	-	-
	10,000–<20,000	7 (21%)	-	-
	20,000–<100,000	6 (18%)	-	-
	≥100,000	3 (9%)	-	-
Years in operation (suppliers only) ^6^	<10	13 (41%)	-	-
	10–30	9 (28%)	-	-
	>30	10 (31%)	-	-
Number of water sources (surveillance only) ^7^	<100	-	2 (6%)	-
	100–<1000	-	10 (30%)	-
	1000–<10,000	-	16 (48%)	-
	≥10,000	-	5 (15%)	-
Number of staff involved in water testing ^8^	<5	20 (67%)	10 (29%)	30 (46%)
	5–<10	3 (10%)	8 (23%)	11 (17%)
	10–<25	6 (20%)	10 (29%)	16 (25%)
	≥25	1 (3%)	7 (20%)	8 (12%)
Annual water monitoring budget (USD)	Total, median ^9^ (interquartile range)	9600 (2200–22,000)	3400 (900–11,000)	7500 (1200–18,000)
Annual water monitoring budget (USD)	Per person served, median ^9^ (interquartile range)	0.056 (0.018–0.131)	0.011 (0.004–0.032)	0.024 (0.006–0.081)
Independently regulated ^10^	Yes	14 (38%)	0 (0%)	14 (19%)
	No	23 (62%)	35 (100%)	58 (81%)

**^1^** Includes national applications and also towns/regions that are part of a national institution; **^2^** Regional labs are affiliated with Ministry of Health in Ethiopia (*n* = 3), Ministry of Water and Energy in Ethiopia (*n* = 1) or Ministry of Water and Environment in Uganda (*n* = 1); **^3^** Data missing for three institutions on population; **^4^** Data missing for two institutions on types of water sources; **^5^** Data not provided for national suppliers with multiple schemes (*n* = 4); **^6^** Data missing for five institutions on years in operation; **^7^** Includes a mix of boreholes, wells, piped water, surface water, *etc.*; data missing for two institutions; **^8^** Data missing for seven institutions on water quality staffing; **^9^** If institutions did not have a specific water quality monitoring budget (*n* = 9), the budget was taken as zero. If these institutions were excluded from the analysis, the median total budgets would be USD 10,000 for suppliers, USD 6000 for surveillance agencies, and USD 8900 total; per person this would be USD 0.067 for suppliers, USD 0.012 for surveillance agencies, and USD 0.039 for total; **^10^** Countries with independent regulators included Kenya (Water Services Regulatory Board (WASREB)), Zambia (National Water Supply and Sanitation Council (NWASCO)), Ghana (Public Utilities Regulatory Commission (PURC)), Tanzania (Energy and Water Utilities Regulatory Authority (EWURA)).

**Table 3:** Water samples, classified by source type.

Water Sample Type	Supplier (*n* = 22,957)	Surveillance (*n* = 1081)	Total (*n* = 24,038)
Network (consumer taps)	15,303 (67%)	338 (31%)	15,641 (65%)
Service Reservoir	3593 (16%)	12 (1%)	3605 (15%)
Water Treatment Plant	1371 (6%)	8 (1%)	1379 (6%)
Incoming water to distribution system ^1^	1364 (6%)	7 (1%)	1371 (6%)
Raw Water	882 (4%)	30 (3%)	912 (4%)
Standpipe/Kiosk (public or commercial taps)	362 (2%)	6 (1%)	368 (2%)
Storage Container/Tank	1 (<1%)	167 (15%)	168 (1%)
Well	2 (<1%)	93 (9%)	95 (<1%)
Borehole	2 (<1%)	77 (7%)	79 (<1%)
Spring	0 (<1%)	25 (2%)	25 (<1%)
Other ^2^	2 (<1%)	7 (1%)	9 (<1%)
Unknown	75 (<1%)	311 (29%)	386 (2%)

**^1^** Incoming water to distribution system = water entering a distribution network, both treated and untreated, and samples from pumping/booster stations; **^2^** Other = includes vended, sachet, recreational water, wastewater, river/stream/lake water, and rainwater.

**Table 4:** Sampling Strategies.

Sampling Strategy	Description	Suppliers (*n* = 19) ^1^	Surveillance (*n* = 13) ^2^
Distribution system	Sampling system components, including raw water, treatment plant water, and piped network water	10	0
Geographic	Sampling an area of jurisdiction that may contain multiple source types	6	0
Population-based	Sampling the sources serving areas with the highest population densities	6	2
Risk-based	Sampling areas where disease rates are known to be high	3	2
Reactive	Sampling after a disease outbreak or after a distribution problem is resolved	3	7
New points	Sampling after a new water source is installed or a new line is laid,	1	3
Ad-hoc	Sampling points determined by the sample collector	2	3

**^1^** Nine suppliers used >1 strategy; **^2^** Four surveillance institutions used >1 strategy.

**Table 5:** Water quality testing practices among surveyed institutions.

Water Testing Characteristic	Category	Supplier (*N* = 37)	Surveillance (*N* = 35)	Total (*N* = 72)
Reported (physical, chemical, and/or microbial) ^1^	Both internal and external lab	25 (68%)	17 (49%)	42 (58%)
	Internal only	11 (30%)	6 (17%)	17 (24%)
	External lab only	1 (3%)	11 (31%)	12 (17%)
	No current testing	0 (0%)	1 (3%)	1 (1%)
Reported regular monitoring	Microbial	27 (77%)	27 (73%)	54 (75%)
	Physical	28 (80%)	12 (32%)	40 (56%)
	Chemical	30 (81%)	14 (38%)	44 (61%)
Types of tests conducted internally	Microbial tests	25 (68%)	19 (54%)	44 (61%)
	Turbidity	29 (78%)	12 (34%)	41 (57%)
	Chlorine	31 (84%)	7 (20%)	38 (53%)
Reported any microbial water testing in the past year ^2^	Yes	34 (92%)	26 (76%)	60 (85%)
	No	2 (8%)	8 (24%)	11 (15%)
Microbial testing indicators ^3^	*E. coli*	12 (32%)	5 (14%)	17 (24%)
	Thermotolerant (fecal) coliforms	15 (41%)	11 (31%)	26 (36%)
	Total coliforms	12 (32%)	9 (26%)	21 (29%)
	Heterotrophic bacteria	8 (23%)	0 (0%)	8 (11%)
	Fecal *Streptococci*	4 (11%)	0 (0%)	4 (6%)
	*Enterococci*	1 (3%)	0 (0%)	1 (1%)
	*Clostridium perfringens*	4 (11%)	0 (0%)	4 (6%)
	H_2_S	1 (3%)	2 (6%)	3 (4%)
	Unspecified	3 (9%)	12 (32%)	15 (21%)
Microbial testing method used ^3^	Membrane filtration	18 (49%)	15 (43%)	33 (46%)
	Most probable number (MPN)	8 (22%)	3 (9%)	11 (15%)
	Presence-absence	10 (27%)	8 (23%)	18 (25%)
	Petrifilm**™**	2 (5%)	1 (3%)	3 (4%)
Average number of microbial tests per year ^1^	Mean (SD)	1731 (3369)	196 (524)	996 (2562)
	Median (interquartile range)	250 (66–1430)	49 (5–148)	110 (18–492)

**^1^** Reported by applicants in the written application baseline survey; **^2^** Missing data for 1 surveillance agency; **^3^** Indicators were used at internal or external laboratories. Some institutions testing multiple indicators (*n* = 21) and/or using multiple methods (*n* = 11).

**Table 6:** Associations with monitoring performance, as measured by WHO Guidelines determined by self-reported testing numbers.

Covariates	Category	Suppliers (*N* = 37)			Surveillance (*N* = 33) ^1^			Total (*N* = 70) ^1^		
		*N*	%	*p*-Value ^2^	*N*	%	*p*-Value ^2^	*N*	%	*p*-Value ^2^
Institution Type	Supplier	20/37	54%	-			-	20/37	54%	<0.01
	Surveillance				6/33	18%		6/33	18%	
Population Served ^3^	<500,000	14/31	45%	0.02	1/20	5%	0.02	15/51	29%	0.02
	≥500,000	6/6	100%		5/12	42%		11/18	61%	
Area of jurisdiction	City/town/district	6/19	32%	0.01	3/25	12%	0.07	9/44	20%	<0.01
	Regional	3/3	100%		1/5	20%		4/8	50%	
	National	11/15	73%		2/3	67%		13/18	72%	
Region	West Africa	9/10	90%	0.01	3/4	75%	0.01	12/14	86%	<0.01
	East/Southern Africa	11/27	41%		3/29	10%		14/56	25%	
Number of connections (suppliers only)	<10,000	4/17	24%	<0.01	-	-	-	4/17	24%	<0.01
	≥10,000	12/16	75%		-	-	-	12/16	75%	
Number of water sources (surveillance only)	<1000	-	-	-	5/13	38%	0.03	5/13	38%	0.05
	≥1000	-	-	-	1/20	5%		1/20	5%	
Urban/rural	Urban	20/37	54%	-	2/7	26%	0.38	22/44	50%	<0.01
	Rural	0/0	0%		4/26	15%		4/26	15%	
Water quality budget per person served (USD)	<$0.05	4/17	24%	<0.01	5/26	19%	0.62	9/43	21%	<0.01
	≥$0.05	16/20	80%		1/7	14%		17/27	63%	
Water quality staff per 10,000 people served ^4^	<1	9/23	39%	0.07	6/25	25%	0.24	15/48	32%	0.49
	≥1	5/6	83%		0/6	0%		5/12	42%	
Years in operation (suppliers only) ^5^	<10	4/13	31%	0.18	-	-		4/13	31%	0.18
	10–30	3/9	44%		-	-		3/9	44%	
	>30	7/10	70%		-	-		7/10	70%	
Independently regulated ^6^	Yes	8/14	57%	0.52	0/0	-	N/A	8/14	57%	0.08
	No	12/23	52%		6/33	18%		18/56	32%	
National standards documented ^7^	Yes	18/35	31%	0.29	3/17	18%	0.64	21/52	40%	0.34
	No	2/2	100%		3/16	19%		5/18	28%	

**^1^** data missing for two surveillance agencies due to a lack of number of sources (1) and a lack of number of reported tests (1); **^2^** *p*-values were calculated using Χ^2^ tests, except when subsets of the populations were examined (suppliers or surveillance only); then Fishers exact test was used due to the small sample size; **^3^** data missing for one surveillance agency on population; **^4^** data missing for eight suppliers and two surveillance agencies on staff numbers; **^5^** data missing for five suppliers on years in operation; **^6^** Countries with independent regulators included Kenya (Water Services Regulatory Board (WASREB)), Zambia (National Water Supply and Sanitation Council (NWASCO)), Ghana (Public Utilities Regulatory Commission (PURC)), Tanzania (Energy and Water Utilities Regulatory Authority (EWURA)); **^7^** Institutions that reported using WHO Guidelines as proxies for national standards were classified as “no” if this was not formally documented.

## Supplementary Materials

The following are available online at www.mdpi.com/1660-4601/13/3/275/s1, Figure S1: Application of Benford’s law to turbidity data from 15 laboratories, Figure S2: Reported water quality testing numbers compared to retrospective datasets, Table S1: Water quality monitoring standards for applicant countries and WHO guidelines, Table S2: Comparing Kenyan applications to national averages, for our eight Kenyan applicant counties (includes suppliers and surveillance agencies), Table S3: Comparing Kenyan applicants to national averages, for Kenyan suppliers.

## Abbreviations

The following abbreviations are used in this manuscript:

AfWA: African Water Association
H_2_S: hydrogen-sulfide producing bacteria
IWA: International Water Association
MfSW: Monitoring for Safe Water
WHO: World Health Organization
