# Supplementary material for: To What Extent is Drinking Water Tested in Sub-Saharan Africa? A Comparative Analysis of Regulated Water Quality Monitoring
Source: Int J Environ Res Public Health. 2016 Mar 2;13(3):275. doi: 10.3390/ijerph13030275 (PMC4808938; doi:10.3390/ijerph13030275)
Supplement: Supplementary file 1 [file ijerph-13-00275-s001.pdf]

Supplementary Materials: To What Extent is Drinking Water Tested in Sub-Saharan Africa? A Comparative Analysis of Regulated Water Quality Monitoring

Rachel Peletz, Emily Kumpel, Mateyo Bonham, Zarah Rahman and Ranjiv Khush

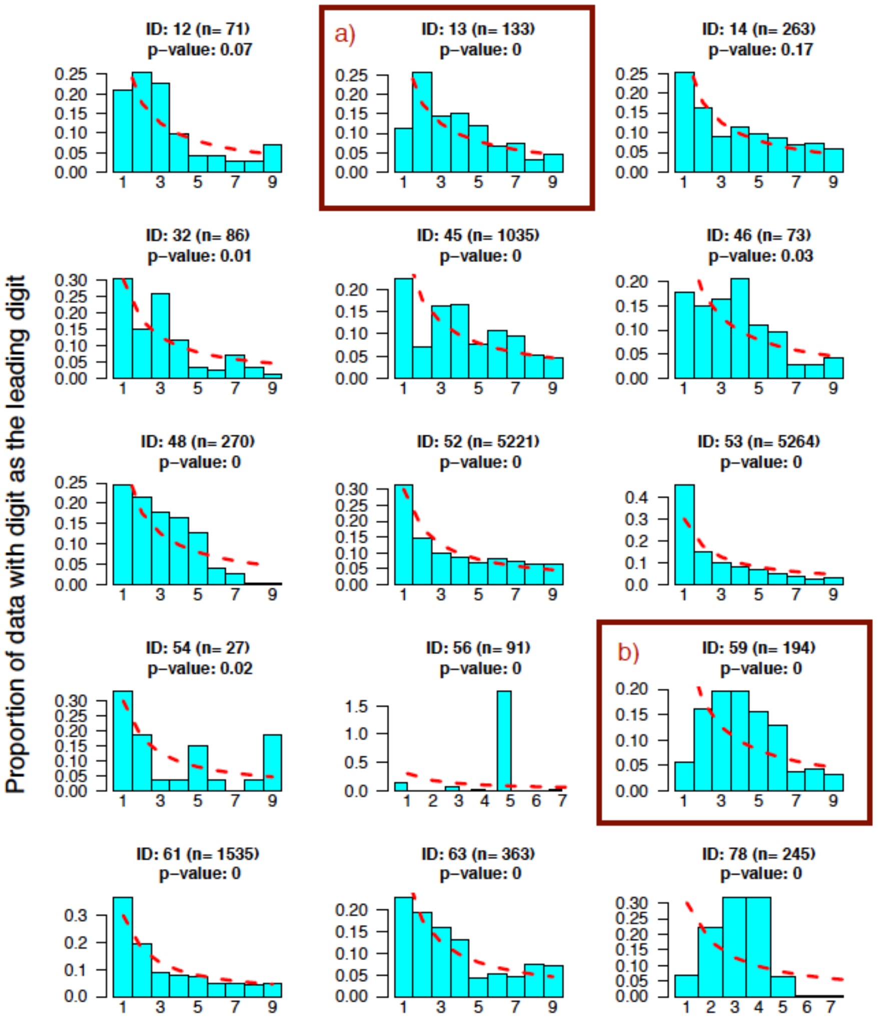

Figure S1. Application of Benford's law to turbidity data from 15 different laboratories. We disaggregated the datasets according to the laboratory that had conducted the tests and evaluated all testing results (regardless of testing date) for water quality parameters that included at least 25 test results that were unbounded for at least two orders of magnitude (e.g., pH was excluded as it ranges from 0–14). Parameters that met these conditions included turbidity, conductivity, fecal coliforms, heterotrophic plate counts at 24 and 48 h, total coliforms, thermotolerant total *coliforms* (TTC), *E. coli*, and *Enterococci*. We were able to examine 55,567 test results from 35% (41/116) of the laboratories, which represented datasets from 67% (23/34) of institutions. In the graph above, the dashed red line represents Benford's distribution and the blue histograms represent the distribution of measured turbidity data. The *p*-values result from a chi-squared test for whether there is a significant difference between the distribution of the testing data and the expected Benford's distribution (*i.e.*, a *p*-value > 0.05 is interpreted as the testing data distribution and Benford's distribution are not significantly different). Plots (a) and (b) show examples of turbidity data that were labeled as suspicious since they both have a *p*-value < 0.05, a large sample size, and their deviance from Benford's law could not explained by the testing method.

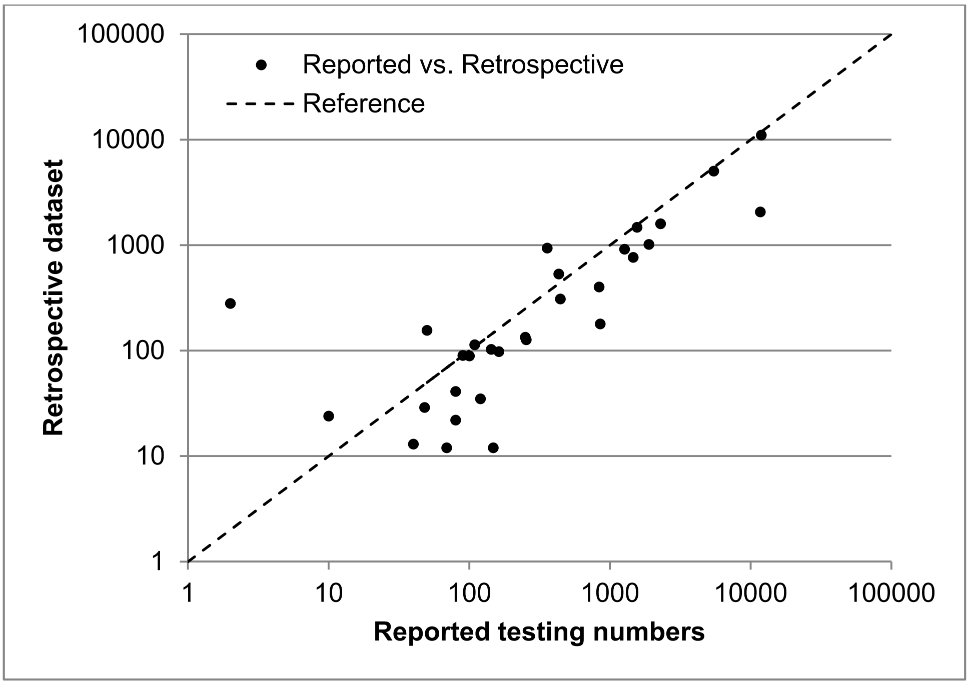

Figure S2. Reported water quality testing numbers compared to retrospective datasets. The dots represent reported testing numbers plotted against retrospective datasets testing numbers for 33 institutions. The dashed line is included for reference if the reported and retrospective numbers were equal. Retrospective dataset numbers were lower than reported numbers for 25/33 (76%) institutions; however, 29/33 (88%) institutions were within one order of magnitude.

Table S1. Water quality monitoring standards for applicant countries and WHO guidelines. This table is included to provide evidence for calculations; the details and complexities of the policies are not captured in this table. For some countries, testing frequency information was only available in the form of "Guidelines", which may have limited enforceability.

**Country**	**Water Suppliers (Piped Networks) by Population**											**Surveillance Agencies (Non-Piped Sources)**	**Reference**
	**<200**	**201–1000**	**1001–2500**	**2501–5000**	**5001–10,000**	**10,001–20,000**	**20,001–25,000**	**25,001–50,000**	**50,001–100,000**	**100,001–500,000**	**>500,000**		
WHO	1 per month				1 sample per 5000 population per month					1 per 10,000 population plus additional 10 samples per month	1 per 50,000 plus an additional 50 samples per month	3–5 year progressive sampling of all point sources	[1]
Benin	2 samples per year	1 sample per month	4 samples per month		5–100 samples per month					110 samples per month		NA (no MfSW applicants)	[2]
Burkina Faso	Use WHO guidelines												[3]
Ethiopia	1 sample per month				1 sample per 5000 population per month					1 sample per 10,000 population plus 10 additional samples per month		Use WHO guidelines	[4,5]
Ghana	1 sample per month				1 sample per 5000 population per month					1 sample per 10,000 population plus 10 additional samples per month		At least 2 tests per year of Total Coliform; but only for 5 years and then further only if changes in area or lack of compliance	[6,7]
Guinea	Use WHO guidelines												[3]
Kenya [1]	1 sample per month				1 sample per 5000 population per month					1 sample per 10,000 population plus 10 additional samples per month	1 sample per 100,000 population plus 15 additional samples per month	WHO guidelines	[8]
Senegal	Use WHO guidelines												[3]

Table S1. *Cont.*

Country	Water Suppliers (Piped Networks) by Population											Surveillance Agencies (Non-Piped Sources)	Reference
	<200	201–1000	1001–2500	2501–5000	5001–10,000	10,001–20,000	20,001–25,000	25,001–50,000	50,001–100,000	100,001–500,000	>500,000		
Tanzania	1 sample per 5000 population per month (max interval between samples = 1 month)						1 sample per 5000 population per month (max interval between samples = 2 weeks)		1 sample per 5000 population per month (max interval between samples = 4 days)	1 sample per 10,000 population per month (max interval between samples = 1 day)		For populations up to 1000: every 6 months for boreholes (>8 m), every 2 months for wells (<8 m), every month for surface water/springs. For populations up to 2000: every 4 months for boreholes, every 1 month for wells, every 2 weeks for surface water/springs. For populations up for 5000, every 3 months for boreholes, every 1 month for wells, every 2 weeks for surface water/springs.	[9]
Uganda	1 sample per month			2 samples per month		3 samples per month		10 samples per month		10 samples every month per 100,000 of population served		Based on population, same as for suppliers	[10,11]
Zambia	NWASCO formula—based on volume produced for utilities: 12 + 1 for each additional 30,000 m^3 above 240,000 m^3 12 is minimum tests per year											No official standards	[11,12]

[1] Testing frequency is also given by volume of water produced in addition to population (which is the same as listed for Zambia).

Table S2. Comparing Kenyan applicants to national averages, for our eight Kenyan applicant counties (includes suppliers and surveillance agencies) [13].

Applicant Counties	% Below Poverty Line	Mean Household Expenditure [1]	% Works for Pay	Primary Education	Improved Water	Improved Sanitation
Isiolo	65%	3.0	17%	36%	59%	40%
Kiambu	24%	5.1	33%	48%	75%	80%
Kisii	51%	2.9	14%	55%	51%	64%
Kisumu	40%	4.4	25%	57%	54%	57%
Nakuru	34%	4.0	30%	55%	60%	76%
Nairobi	22%	7.2	47%	38%	84%	88%
Samburu	71%	1.9	10%	26%	34%	20%
Turkana	88%	1.4	6%	15%	39%	8%
Applicant county average (8 counties)	51%	3.6	22%	41%	54%	52%
Kenya average (47 counties)	45%	3.4	24%	52%	53%	61%

[1] Mean household expenditure (in thousand KES) per adult equivalent per month.

Table S3. Comparing Kenyan applicants to national averages, for Kenyan suppliers [14].

Applicant Suppliers	Total Number of Connections	Turnover (KES Million)	Production m^3 (000)	Non-Revenue Water
Nairobi	472,205	7227	190,445	38
Nakuru	48,157	604	12,434	46
Kisumu	27,347	415	8893	47
Gusii	16,339	95	2060	47
Ruiru Juja	9275	98	1383	30
Isiolo	7441	54	1093	43
Lodwar	5238	41	1060	37
Maralal	1957	10	299	38
Applicant supplier average (8 applicants)	73,495	1068	27,208	41
Applicant supplier average, excluding Nairobi Water (7 applicants)	16,536	188	3889	41
Kenya average (65 suppliers)	19,554	228	5583	42

References

1. WHO. *WHO Guidelines for Drinking-Water Quality*, 4th ed.; WHO: Geneva, Switzerland, 2011.
2. Republique du benin. *Fixant les Procedures de Delimitation des Perimetres de Protection (Decret No 2011-094)*; Presidence de la Repulique: Cotonou, Benin, 2001. (In French)
3. L'Office national de l'eau et de l'assainissement (ONEA) (Ouagadougou, Burkina Faso); Société des Eaux de Guinée (SEG) (Conakry, Guinea); Service National de l'Hygiène (SNH). Personal communication, 2013.
4. Ethiopian Standards Agency. *Drinking Water—Speficications (ES 261:2001)*; Quality and Standards Authority of Ethiopia: Addis Ababa, Ethiopia, 2001.
5. Ethiopian Ministry of Health. *National Drinking Water Quality Monitoring and Surveillance Strategy*; Ethiopian Ministry of Health: Addis Ababa, Ethiopia, 2011.
6. Government of Ghana. *Community Water and Sanitation Agency Water Safety Framework*; Government of Ghana Ministry of Water Resources, Works and Housing: Accra, Ghana, 2010.

7. Ghana Standards Authority. *Water. Quality—Specification for Drinking Water (GS 175-1: 2013)*, 4th ed.; Ghana Standards Authority: Accra, Ghana, 2013.
8. WASREB. *Drinking Water Quality and Effluent Monitoring Guidelines*; WASREB: Nairobi, Kenya, 2009.
9. The United Republic of Tanzania Ministry of Water. *Water Sector Development Programme 2006–2025: Programme Implementation Manual*; United Republic of Tanzania Ministry of Water: Dar es Salaam, Tanzania, 2011.
10. Uganda National Bureau of Standards. *Uganda Standard: Drinking (Potable) Water—Specification*; Uganda National Bureau of Standards: Kampala, Uganda, 2008.
11. Zambian Ministry of Health (Lusaka, Zambia); Uganda Ministry of Health (Kampala, Uganda). Personal communication, 2013.
12. NWASCO. *Guidelines on Water Quality Monitoring*; NWASCO: Lusaka, Zambia, 2010.
13. Njonjo, K.S. *Exploring Kenya's Inequality: Pulling Apart or Pooling Together? Abridged Report*; Kenya National Bureau of Statistics and the Society for International Development—East Africa: Nairobi, Kenya, 2013.
14. WASREB. *WASREB 2014 Impact Report: A Performance Review of Kenya's Water Services Sector 2011–2012*; WASREB: Nairobi, Kenya, 2014.

© 2016 by the authors; licensee MDPI, Basel, Switzerland. This article is an open access article distributed under the terms and conditions of the Creative Commons by Attribution (CC-BY) license (http://creativecommons.org/licenses/by/4.0/).
